# The trajectory of high sensitivity C-reactive protein is associated with incident diabetes in Chinese adults

**DOI:** 10.1186/s12986-020-00472-w

**Published:** 2020-06-30

**Authors:** Renying Xu, Xiaomin Jiang, Zhuping Fan, Yanping Wan, Xiang Gao

**Affiliations:** 1 Department of Clinical Nutrition, Ren Ji Hospital, School of Medicine, Shanghai Jiao Tong University, Shanghai, 200127 China; 2 Shanghai Key Laboratory of Pediatric Gastroenterology and Nutrition, Shanghai, China; 3 Department of Digestion, Ren Ji Hospital, School of Medicine, Shanghai Jiao Tong University, Shanghai, China; 4 Department of Nutritional Sciences, The Pennsylvania State University, University Park, PA USA

**Keywords:** Trajectory, High sensitivity C-reactive protein (hs-CRP), Diabetes

## Abstract

**Background:**

We performed a cohort study to evaluate the association between the CRP trajectory and incident diabetes in Chinese adults.

**Methods:**

Included were 6439 adults (4111 men and 2249 women; aged 46.6 ± 11.9 years). The concentration of high sensitivity CRP (hs-CRP) was measured in 2013 (baseline), 2014, and 2015. The hs-CRP trajectory was identified based the above three measurements by latent mixture modeling. Incident diabetes cases were diagnosed by fasting blood glucose (≥126 mg/dl) or Hb A1c (≥6.5%) during subsequent 3 years (2016–2018).

**Results:**

Hs-CRP concentration during 2013–2015 was classified into 3 levels: low (< 1.0 mg/L), moderate (1.0–3.0 mg/L), and high (≥3.0 mg/L) based on a statement by American Heart Association. We named four hs-CRP trajectories as following: “low-stable” (low in 2013 and maintained at low concentration in 2014 and 2015), “moderate-fluctuated” (moderate in 2013, then increased to high concentration in 2014, and decreased to low concentration in 2015), “high-decreased” (high in 2013 but decreased to moderate concentration in 2014 and 2015), and “moderate-increased (moderate in 2013 and increased to high concentration in 2014 and 2015)”. We identified 235 incident diabetes during subsequent 3 years. The adjusted HR for incident diabetes was 1.71 (95% CI: 1.02, 2.87) comparing the moderate-increased and the low-stable group, after adjusting for potential confounders. In the secondary analyses, two single-measured hs-CRP concentration (in 2013 or in 2015) and the average of hs-CRP were associated with high risk of diabetes (P-trend< 0.01 for all).

**Conclusions:**

The hs-CRP trajectory pattern was associated with altered incident diabetes in Chinese adults.

## Background

It is estimated that people with diabetes will reach an alarming number of 366 million in 2030 globally [1]. Diabetes causes a series of microvascular and macrovascular changes [2], thus leads the increased prevalence of disability and mortality and throws a heavy burden to human health.

One of the most important strategies to curb the dramatic increase of diabetes is to identify people with high risk for diabetes and to implement early intervention. C-reactive protein (CRP), a classical inflammatory biomarker, has been considered as an indicative parameter for diabetes and related complications in both cross-sectional [3–5] and longitudinal studies [6–10]. However, almost all the above-mentioned studies focused on baseline measure of CRP and neglected long-term change. The concentration of CRP changes in life [11]. Not taken time-varying and cumulative average over time into consideration might may lead to misclassification of CRP status and result in confuses [12]. For example, one clinical trial suggested that CRP may not be an optimal factor to predictive changes in cardiovascular risk among diabetic patients [13] while another cohort study did not find the association between baseline CRP and incident diabetes over thirteen-year follow up [14].

Based on repeated variable, trajectory analysis taking its advantage over traditional analysis lies in its unique ability to record long-term change over time. Thus, the trajectory approach could account for the dynamic natural change overtime and facilitate to identify a cluster of individuals who have similar longitudinal change patterns of CRP [15, 16]. Therefore, the aim of this study was to prospectively evaluate the association between the CRP trajectory during 2013–2015 and subsequent risk of diabetes, as assessed by both fasting blood glucose (FBG) and glycated hemoglobin A1c (HbA1c), in approximately 6300 Chinese adults. As secondary analyses, we also examined the association between baseline (2013), and cumulative average of high sensitivity CRP (hs-CRP) and future diabetes risk.

## Methods

### Study population

All the participants were recruited from Health Management Center from January 1, 2013 to October 31, 2018. A total number of 55,155 adults with measurements of FBG, HbA1c and hs-CRP was eligible for the study. The concentration of hs-CRP was measured at 2013 (baseline), 2014, and 2015 and the trajectory was identified based on the three measurements. HbA1c and FBG were repeatedly annually measured during 5 years of follow up. We excluded participants with history of diabetes, high HbA1c (≥ 6.5%), high fasting blood glucose (≥7.0 mmol/L) or extremely high concentration of hs-CRP (≥10 mg/L) during 2013–2015, and those lost to follow up. Because hs-CRP status is strongly influenced by presence of cardiovascular disease, cancer and major metabolic disorders (hypertension, dyslipidemia and hyperuricemia), we further excluded participants with these conditions. Finally, included were 6349 adults (4111 men and 2238 women; 18–89 years old) in the analysis (Supplemental Fig. 1). Participants included in the study were younger, with higher proportion of women, and lower concentration of hs-CRP, FBG, and HbA1c at baseline, compared with those were not included (Supplemental Table 1). The study protocol was approved by the Ethical Committee of Ren Ji Hospital, School of Medicine, Shanghai Jiao Tong University. As a de-identified secondary data analysis, patients’ consent was waived by the Ethical Committee.

### Assessment of incident diabetes

Venous blood samples were drawn and transfused into vacuum tubes containing EDTA in the morning after participants were fasted for 6 hours. The whole blood was stored at 4 °C for further analysis. The level of HbA1c was measured by high performance liquid chromatography, using the fully automated VARIANT™ II Hemoglobin Testing System (Bio-Rad, U.S). The measurement range was between 2.0 and 18.0%. An automatic analyzer (Roche 701 Bioanalyzer, Roche, UK) was used to measure FBG with the hexokinase/glucose-6-phosphate dehydrogenase method. The coefficient of variation using blind quality control specimens was 2.0%. Diabetes was confirmed if FBG (≥7.0 mmol/L = 126 mg/dl) or HbA1c (≥6.5%) [17, 18].

### Measurement of hs-CRP and other biochemical parameters

The concentration of hs-CRP was measured by immunotubidimetric method (CardioPhase hsCRP kit, Siemens Healthcare Diagnostics Products GmbH, German). The lower limit of detection was 0.01 mg/L. The intraassay CV was 7.6% and the interassay CV was 4.0%. Hs-CRP concentration during 2013–2015 was classified into 3 categories: low (< 1.0 mg/L), moderate (1.0–3.0 mg/L), and high (≥3.0 mg/L) based on a statement by American Heart Association [19].

The following variables were measured at baseline. Total cholesterol, triglycerides, high-density-lipoprotein cholesterol, low-density- lipoprotein cholesterol, and blood creatinine were measured by enzyme linked immunosorbent assay (Roche 701 Bioanalyzer, Roche, UK). White blood cell (WBC) were also measured. All the measurements were completed in the Clinical Laboratory of Ren Ji Hospital. The eGFR was calculated using the Chronic Kidney Disease Epidemiology Collaboration 2-level race equation [20].

### Assessment of other confounders

Body weight and height were annually measured, and BMI was calculated by body weight (kg) divided by height square (m^**2**^). The latest BMI was calculated as the latest BMI measured before the onset of the diabetes (e.g., if incident diabetes was determined in 2016, then BMI measured in 2015 was used). Blood pressure and history of the diseases were obtained at baseline. Blood pressure was measured twice using an automatic blood-pressure meter (HBP-9020, OMRON (China) Co., Ltd.) after participants were seated for at least 10 min. The average of two measurements was recorded for further analysis. The history of hypertension, diabetes/impaired fasting glucose, dyslipidemia, hyperuricemia, stroke and hemorrhage, and coronary heart diseases (coronary atherosclerosis, coronary artery bypass grafting, stent surgery, and ischemic infarction) collected via a self-report questionnaire **if the participants were diagnosed with these diseases by a physician or they were taking any drugs for these diseases** [21].

### Statistical analysis

Data were presented as mean ± standard deviation. Since hs-CRP was in abnormal distribution, it was square transformed. We completed all statistical analyses by SAS version 9.4 (SAS Institute, Inc., Cary, NC). The correlation between FBG, HbA1c, BMI, blood pressure, lipid profile, and eGFR was evaluated by Spearman correlation analysis. Formal hypothesis testing will be two-sided with a significant level of 0.05. The person-time of follow-up for each participant was determined from January 1, 2016 to either the onset date of diabetes, or the end of follow-up (December 31, 2018), whichever came first. The time unit for the analysis was a month.

The hs-CRP trajectory was identified by **PROC TRAJ** procedure based on three measurement obtained in 2013, 2014, and 2015 [12, 22]. A basic one group model was fitted with all groups set to a quadratic equation. Then, we fitted a two-group, three-group, three-group, four-group, and five-group model as well. The model with four groups was identified as the best, as suggested by the lowest value of Bayesian information criterion. We then compared the model with different functional forms. In the final model, we had one pattern with a quadratic order term and three patterns with a cubic order term. Further, we calculated the posterior predicted probability for each participant of being a member of each trajectory [15]. The average posterior probability for each trajectory was 0.84, 0.87, 0.85, and 0.83.

In secondary analyses, we used baseline hs-CRP, hs-CRP in 2015, and the average of three measurements of CRP as exposures. Participants were further classified into following groups based on either single assessment of hs-CRP in 2013, in 2015, or the average of hs-CRP during 2013–2015: low-risk (< 1.0 mg/L), intermediate-risk (1.0–3.0 mg/L), and high-risk (≥3.0 mg/L) [19].

We used the proportional hazards Cox model to evaluate the association between the hs-CRP trajectory and incident diabetes. We adjusted for potential confounders in two different models: **model 1**, adjusting for age (y) and sex; and **model 2** further adjusting baseline BMI (kg/m^2^), systolic blood pressure (mmHg), diastolic blood pressure (mmHg), total cholesterol (mmol/L), triglycerides (mmol/L), low-density-lipoprotein cholesterol (mmol/L), high-density-lipoprotein cholesterol (mmol/L), eGFR (ml/min/1.73m^2^), FBG (mmol/L), and HbA1c (%) because these variables were closely associated with both FBG and HbA1c (Supplemental Table 2). In a secondary analysis, we further adjusted for hs-CRP (2013) to understand whether the potential association between hs-CRP trajectory and diabetes risk was driven by the baseline CRP status, although we were aware of the risk of over-adjustment and collinearity.

We tested the interaction between hs-CRP trajectory and sex, age (<65y **vs.** ≥65y) [12], and BMI (< 28.0 **vs.** ≥28.0 kg/m^2^) [23], in relation to incident diabetes.

## Results

In the current study, mean baseline age, square-transformed hs-CRP, and HbA1c was 46.6 ± 11.9 years, 0.93 ± 0.48 mg/L, and 5.4 ± 0.3%, respectively. Baseline characteristics were presented in Table 1.

**Table 1:** Baseline characteristics across different trajectories of hs-CRP among 6349 Chinese adults

Variables	Low-stable	Moderate-increased	Moderate-fluctuated	High-decreased
n	5174	208	679	288
Age, y	46.3 ± 12.1	48.0 ± 12.2	48.3 ± 9.4	47.0 ± 12.8
Sex, women, %	36.0	30.3	33.1	30.2
BMI, kg/m^2^	23.9 ± 3.0	25.9 ± 3.5	23.7 ± 2.7	25.6 ± 3.2
SBP, mmHg	122 ± 16.2	127 ± 15.9	123 ± 12.9	126 ± 16.6
DBP, mmHg	75.9 ± 11.0	80.0 ± 11.8	72.7 ± 9.9	77.2 ± 11.4
FBG, mmol/L	5.1 ± 0.5	5.2 ± 0.6	5.3 ± 0.5	5.2 ± 0.4
HbA1c, %	5.4 ± 0.4	5.5 ± 0.4	5.3 ± 0.3	5.4 ± 0.4
TC, mmol/L	5.0 ± 0.9	5.1 ± 0.9	5.1 ± 0.7	5.0 ± 0.9
TG, mmol/L	1.6 ± 1.3	1.8 ± 1.2	1.4 ± 0.8	1.8 ± 1.1
HDL-C, mmol/L	1.4 ± 0.4	1.2 ± 0.3	1.3 ± 0.3	1.2 ± 0.3
LDL-C, mmol/L	3.0 ± 0.8	3.2 ± 0.8	3.2 ± 0.6	3.1 ± 0.8
eGFR, ml/min/1.73m^2^	113 ± 25.1	109 ± 24.2	133 ± 32.5	117 ± 27.3
WBC, 10^9^/L	6.1 ± 1.5	6.9 ± 1.6	6.2 ± 1.6	7.1 ± 1.8
Hs-CRP, mg/L^a^	0.6 (0.01, 9.9)	2.42 (0.1, 9.4)	0.83 (0.17, 9.9)	3.47 (0.46, 9.95)

Note: 1. Abbreviation: *hs-CRP* High sensitivity C-reactive protein, *BMI* Body mass index, *SBP* Systolic blood pressure, *DBP* Diastolic blood pressure, *FBG* Fasting blood glucose, *HbA1c* Glycated hemoglobin A1c, *TC* Total cholesterol, *TG* Triglyceride, *HDL-C* High density lipoprotein cholesterol, *LDL-C* Low density lipoprotein cholesterol, *eGFR* Estimating glomerular filtration rate, *WBC* White blood cell

2. ^a^, abnormal distribution. The data was shown as medium plus full range

**Fig. 1:**
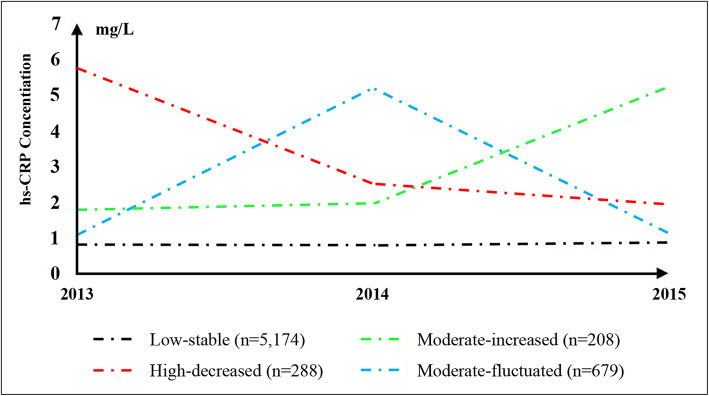
The trajectory of high sensitivity C-reactive protein in 6349 Chinese adults. Hs-CRP level during 2013–2015 was classified into 3 levels: low (< 1.0 mg/L), moderate (1.0–3.0 mg/L), and high (≥3.0 mg/L) based on a statement by American Heart Association. We named four hs-CRP trajectories during 2013–2015 as following: “low-stable” (hs-CRP concentration was low in 2013 and maintained at low concentration in 2014 and 2015), “moderate-fluctuated” (hs-CRP concentration was moderate in 2013, then increased to high concentration in 2014, and decreased to low concentration in 2015), “high-decreased” (hs-CRP concentration was high in 2013 but decreased to moderate concentration in 2014 and 2015), and “moderate-increased (hs-CRP concentration was moderate in 2013 and increased to high concentration in 2014 and 2015)”

We identified four hs-CRP trajectories in the study population: 77.4% (*n* = 5174) of the participants whose baseline hs-CRP concentration was low and maintained stable (referred as “low-stable”), 14.2% (*n* = 679) of the participants whose baseline hs-CRP was moderate but changed dramatically (referred as “moderate-fluctuated”), 5.0% (*n* = 288) of the participants whose baseline hs-CRP were high but decreased to moderate concentration (referred as “high-decreased”), and 3.4% (*n* = 208) of the participants whose baseline hs-CRP was moderate and increased to high concentration (referred as “moderate-increased”) (Fig. 1). The average concentration of hs-CRP in 2013, 2014, and 2015 was presented in Supplemental Table 3.

We identified 235 incident diabetes over subsequent 3 years after 2015. Each standard deviation (≈1.02 mg/L) of cumulative hs-CRP during follow up (2016–2018) was associated with 24% (HR = 1.24, 95% CI: 1.04, 1.47) higher risk of developing diabetes in multivariate-adjusted model. Compared with the low-stable group, moderate-increased group was associated with the highest likelihood of incident diabetes (HR = 1.71, 95% CI: 1.02, 2.87) (Table 2). Further adjusting for baseline hs-CRP (Table 2) did not materially change the association. Similar significant results were observed when we further adjusted for baseline WBC (adjusted HR comparing the two groups was 1.69, 95%CI: 1.002, 2.84). Including the latest BMI in multivariate-adjusted model got similar results with main analysis (Supplemental Table 4).

**Table 2:** Adjusted hazards ratios and 95% confidence intervals for risks of incident diabetes (2016–2018) across different trajectories of hs-CRP during 2013 and 2015 among 6349 Chinese adults

Model	Different change patterns of high sensitivity C-reactive protein			
	Low-stable	Moderate-increased	Moderate-fluctuated	High-decreased
n	5174	208	679	288
Case #	168	16	32	19
Age- and sex-adjusted	**1 (ref)**	2.18 (1.31, 3.64)	1.40 (0.96, 2.05)	1.94 (1.21, 3.12)
Multivariate-adjusted ^a^	**1 (ref)**	1.71 (1.02, 2.87)	1.44 (0.95, 2.17)	1.37 (0.84, 2.22)
Further adjustment for 2013 hs-CRP^a^	**1 (ref)**	1.77 (0.99, 3.17)	1.46 (0.95, 2.24)	1.43 (0.8, 2.54)

**Abbreviation**: *hs-CRP* High sensitivity C-reactive protein

^a^ Adjusted for age, sex, baseline BMI (kg/m^2^), systolic blood pressure (mmHg), diastolic blood pressure (mmHg), total cholesterol (mmol/L), triglyceride (mmol/L), low-density-lipoprotein cholesterol (mmol/L), high-density-lipoprotein cholesterol (mmol/L), eGFR (ml/min/1.73m^2^), fasting blood glucose (mmol/L), and glycated hemoglobin A1c (%)

We did not find the association between high-decreased and moderate-fluctuated trajectories, and risk of incident diabetes. Both baseline and the cumulative average hs-CRP were associated with high risk of incident diabetes (Table 3). We did not find the significant interaction between sex, age, and BMI, in relation to incident diabetes (P-interaction > 0.1 for all).

**Table 3:** The adjusted hazard ratios and 95% confidence interval for incident diabetes across different hs-CRP groups among 6349 Chinese adults

Variables	Model	Low-risk (< 1 mg/L)	Intermediate-risk (1–2.9 mg/L)	High-risk (≥3 mg/L)	Each SD	P trend
Baseline hs-CRP concentration^a^	n	4243	1663	443	–	–
	Case #	157	105	40	–	–
	Model	**1 (ref)**	1.22 (0.94, 1.58)	1.67 (1.17, 2.39)	1.11 (1.01, 1.22)	0.005
Concentration of hs-CRP in 2015^b^	n	4174	1787	388	–	–
	Case #	111	95	29	–	–
	Model	**1 (ref)**	1.44 (1.08, 1.9)	1.94 (1.28, 2.94)	1.19 (1.08, 1.30)	0.001
Cumulative average of hs-CRP concentration^b^	n	5197	995	157	–	–
	Case #	171	52	12	–	–
	Model	**1 (ref)**	1.4 (1.001, 1.94)	1.87 (1.03, 3.39)	1.24 (1.04, 1.47)	0.008

**Note**:

1. **Abbreviation**: *hs-CRP* High-sensitivity C-reactive protein

2. ^a^ based on five years of follow up (2014–2018)

3. ^b^ based on three years of follow-up (2016–2018)

4. Adjusting for age (y) and sex, baseline BMI (kg/m^2^), systolic blood pressure (mmHg), diastolic blood pressure (mmHg), total cholesterol (mmol/L), triglyceride (mmol/L), low-density-lipoprotein cholesterol (mmol/L), high-density-lipoprotein cholesterol (mmol/L), eGFR (ml/min/1.73m^2^), fasting blood glucose (mmol/L), and glycated hemoglobin A1c (%)

## Discussion

In the current study, we observed four hs-CRP trajectories and found that the “moderate-increased” trajectory was associated with subsequent risk of diabetes in about 6300 Chinese adults free of cardiovascular disease, cancer and major metabolic disorders at baseline, after adjusting for potential confounders. Consisted with previous studies [4, 7], we found that both baseline and the average of hs-CRP were associated with incident diabetes. Our results strengthen the concept that inflammation is involved in the etiology of developing diabetes [24].

Hs-CRP has been reported to be associated with both cardiovascular diseases and diabetes in previous studies [25–27]. However, it may not be optimal to evaluate baseline hs-CRP and incident diabetes because baseline hs-CRP could not reflect longitudinal changes in inflammation status. Data were limited to evaluate the association between changes in hs-CRP and incident diabetes. Tabák et al. [11] reported that CRP concentrations increased with time among both incident diabetes cases and controls in a cohort study in 7350 British participants. However, they did not evaluate the association between changes in CRP and the risk of incident diabetes. Another cohort study included 14,228 participants without diabetes and followed them for thirteen years. The concentration of hs-CRP was repeatedly measured. Changes in CRP were found to be associated with increase in HbA1c by linear mix model, but the association between each standard deviation increase in CRP was not associated with annual changes in HbA1c (*p = 0.15*) [28]. Another observational study included 42 patients with type 1 and 94 patients with type 2 diabetes. Two-year changes (2-year follow-up divided by baseline) of CRP was associated with two-year increases of HbA1c [29]. Our study found that individuals with increased hs-CRP concentration during 2013–2015 (i.e., the moderate-increased trajectory) had ~ 70% higher subsequent diabetes risk, relative to those with stably low hs-CRP concentration over 2 years. The association did not materially change after we further adjusted for baseline hs-CRP concentration, suggesting longitudinal change in hs-CRP could be important for diabetes risk. Similar to our study, Parrinello et al. [30] classified participants into four different groups based on two hs-CRP measurements. Compared to persons with sustained low/moderate hs-CRP (≤3 mg/L), those with increased or sustained high hs-CRP (> 3 mg/L) had an increased risk of incident diabetes, whereas those with deceased hs-CRP did not. However, it might be less stable to calculate the trajectory by using two measurements. Further, we found that the high-decreased trajectory was not associated with the risk of incident diabetes.

Strengths of the present study included a large sample size of healthy Chinese adults free of hypertension, diabetes, dyslipidemia, coronary heart diseases, hyperuricemia, stroke, and cancer at baseline. To the best of our knowledge, this is the first study describing the association between the trajectory of hs-CRP and incident diabetes. Four analytical approaches including baseline, the measurement in 2015, cumulative average, and trajectories were analyzed to strengthen the reliability of the results.

Our study has several limitations. First, we did not collect information about history of autoimmune diseases such as inflammatory bowel diseases, systemic lupus erythematosus, celiac disease, glomerulonephritis, and asthma. The diseases themselves could increase the concentration of hs-CRP and its drugs used in these diseases might also increase the concentration of blood glucose as well. However, the incidence of these diseases in general population is low in China [31–35] and the dosage of corticosteroid during the quiescent phage of the diseases is low. We thus assumed the possible impacts of the diseases and its related treatment is minimal in the current study. Further, smoking, diet, and physical activity were not collected and may lead to residual confounding. Although we collected the information on smoking, the self-report prevalence of smoking was very low (1%) compared with about 30% of the current smokers in Chinese adults [36]. We thus did not include smoking variable in the model. Second, information regarding acute inflammation and infection was not collected in the current study. We could not exclude the possibility that high hs-CRP was cause by acute inflammation [26], rather than low-grade systemic inflammation. However, we excluded participants with hs-CRP ≥10 mg/L in the analyses. Further adjusting WBC also did not change the association. Then, we did not collect information about diabetes mellitus-related medications, which were reported to be associated with hs-CRP [37, 38]. However, we excluded patients whose FBG ≥ 7.0 mmol/L or HbA1c ≥ 6.5% at baseline, which might mitigate the distraction because these participants were most likely to receive such treatment. Information on metabolic diseases was self-reported and we cannot exclude the possibility of recall bias, thus some participants with metabolic diseases might remain in the study. Further, incident diabetes was identified by FBG and postprandial hyperglycemia was not taken into consideration, which might result in underdiagnosis of diabetes cases. However, it is acceptable to identify diabetes cases by FBG in some of epidemiological studies [14, 39]. Third, we did not review medical records and the onset of the diseases was assessed at the visit time. However, the FBG and HbA1c examination has been conducted every year. Diabetes confirmed by single measurement of FBG or HbA1c, but had not further been assessed by oral glucose tolerance test, might lead to misclassification of diabetes status. Fourth, we did not consider seasonal fluctuations in FBG and HbA1c [40, 41], and thus we could exclude the possibility of misdiagnosis of incident cases of diabetes. Finally, study population was recruited from Healthy Examination in our hospital, which could not represent of general population. Prospective studies with representative population, and deliberately collection of information about potential confounders, and long follow up are warranted to confirm our results in the future.

## Conclusion

The moderate-increased trajectory of hs-CRP was associated with incident diabetes in Chinese adults.

## Supplementary information

**Additional file 1: Supplemental Figure 1.** Sample recruitment. Coronary heart diseases include coronary atherosclerosis, coronary artery bypass grafting, stent surgery and ischemic infarction. **Abbreviation**: **hs-CRP**, high sensitivity C-reactive protein; **HbA1c**, glycated hemoglobin A1c; **IFG**, impaired fasting glucose; **FBG**, fasting blood glucose. **Supplemental Table 1.** Baseline characteristics between participants remained and out of the study. **Supplemental Table 2.** The correlation between fasting blood glucose, glycated hemoglobin A1c, body mass index, blood pressure, lipid profile, and estimated glomerular filtration rate in 6349 Chinese adults. **Supplemental Table 3.** The mean and standard deviation at three time points across different trajectories of hs-CRP (mg/L). **Supplemental Table 4.** Adjusted hazards ratios and 95% confidence intervals for risks of incident diabetes (2016–2018) across different trajectories of hs-CRP during 2013 and 2015 among 6349 Chinese adults.

## Data Availability

All the datasets and SAS code are available upon readers’ request.

## Abbreviations

CRP: C-reactive protein
eGFR: Estimated glomerular filtration rate
FBG: Fasting blood glucose
HbA1c: Glycated hemoglobin A1c
Hs-CRP: High sensitivity C-reactive protein
