# Supplementary material for: The trajectory of high sensitivity C-reactive protein is associated with incident diabetes in Chinese adults
Source: Nutr Metab (Lond). 2020 Jun 30;17:49. doi: 10.1186/s12986-020-00472-w (PMC7325292; doi:10.1186/s12986-020-00472-w)
Supplement: Supplementary file 1 — Additional file 1: Supplemental Figure 1. Sample recruitment. Coronary heart diseases include coronary atherosclerosis, coronary artery bypass grafting, stent surgery and ischemic infarction. Abbreviation: hs-CRP, high sensitivity C-reactive protein; HbA1c, glycated hemoglobin A1c; IFG, impaired fasting glucose; FBG, fasting blood glucose. Supplemental Table 1. Baseline characteristics between participants remained and out of the study. Supplemental Table 2. The correlation between fasting blood glucose, glycated hemoglobin A1c, body mass index, blood pressure, lipid profile, and estimated glomerular filtration rate in 6349 Chinese adults. Supplemental Table 3. The mean and standard deviation at three time points across different trajectories of hs-CRP (mg/L). Supplemental Table 4. Adjusted hazards ratios and 95% confidence intervals for risks of incident diabetes (2016–2018) across different trajectories of hs-CRP during 2013 and 2015 among 6349 Chinese adults. [file 12986_2020_472_MOESM1_ESM.docx]

**
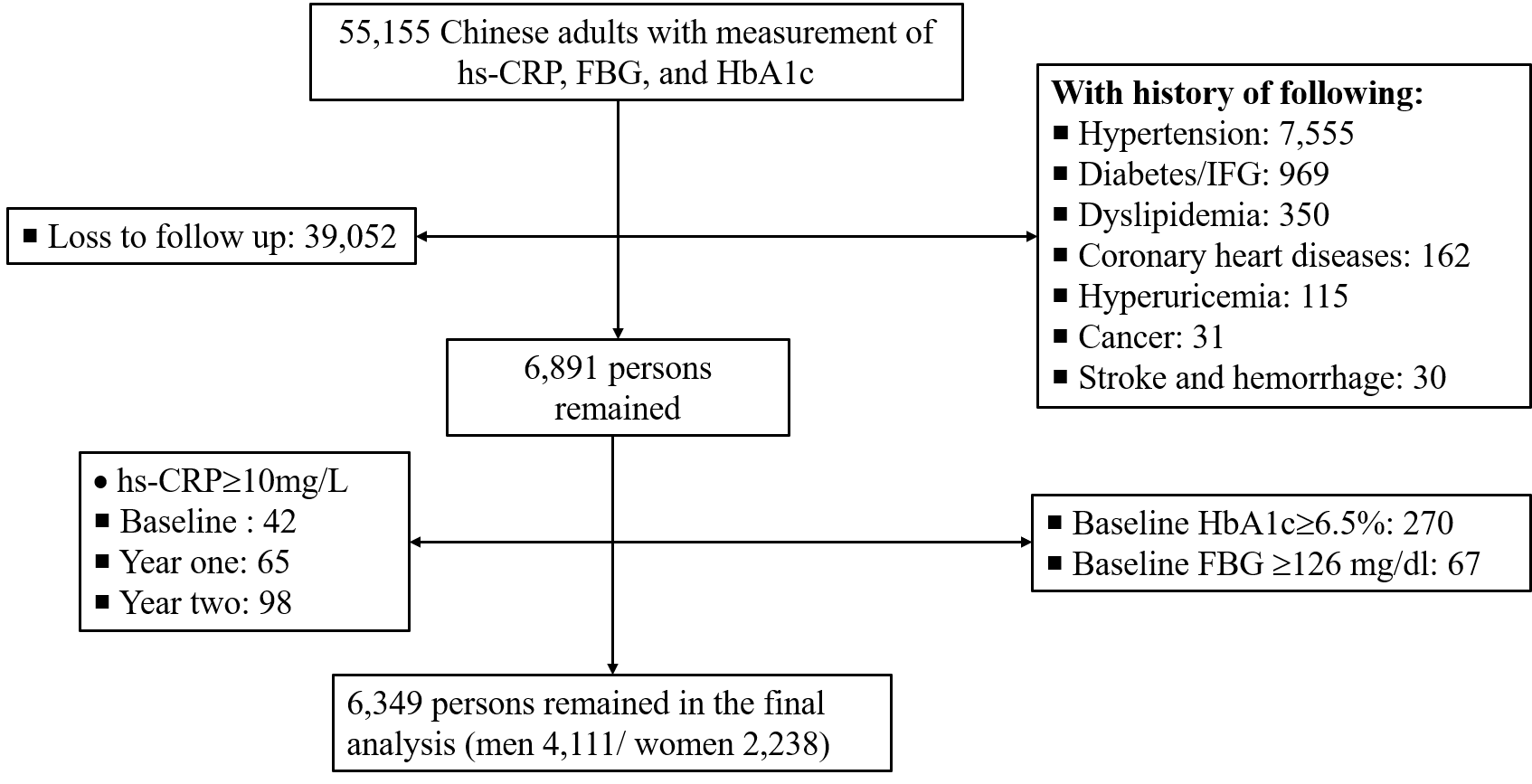
**

**Supplemental Figure 1**. Sample recruitment. Coronary heart diseases include **coronary atherosclerosis,** coronary artery bypass grafting, stent surgery and ischemic infarction. **Abbreviation**: **hs-CRP**, high sensitivity C-reactive protein; **HbA1c**, glycated hemoglobin A1c; **IFG**, impaired fasting glucose; **FBG**, fasting blood glucose.

**Supplemental Table 1**. Baseline characteristics between participants remained and out of the study

| Variables | Participants in the study | Participants out of the study | P value |
| --- | --- | --- | --- |
| Sample | 6, 349 | 48,806 | -- |
| Age, y | 46.8±11.7 | 50.5±15.0 | <0.001 |
| Sex, women, % | 35.4 | 42.2 | <0.001 |
| hs-CRP, mg/L* | 0.93±0.49 | 1.02±0.74 | <0.001 |
| BMI, kg/m^2^ | 24.0±3.0 | 24.2±3.4 | <0.001 |
| SBP, mmHg | 122.7±15.7 | 127.8±19.4 | <0.001 |
| DBP, mmHg | 75.6±11.0 | 76.6±12.5 | <0.001 |
| FBG, mmol/L | 5.2±0.6 | 5.4±1.3 | <0.001 |
| HbA1c, % | 5.4±0.4 | 5.6±0.8 | <0.001 |
| TC, mmol/L | 5.0±0.9 | 5.0±1.0 | 0.17 |
| TG, mmol/L | 1.6±1.2 | 1.6±1.4 | <0.001 |
| HDL-C, mmol/L | 1.3±0.4 | 1.4±0.4 | <0.001 |
| LDL-C, mmol/L | 3.0±0.8 | 3.0±0.8 | 0.01 |
| eGFR, ml/min/1.73m^2^ | 116.1±27.2 | 110.2±33.9 | <0.001 |
| WBC, 10^9^/L | 6.2±1.5 | 6.2±1.6 | 0.89 |

**Note**:

1. Abbreviation: **hs-CRP**, high sensitivity C-reactive protein; **HbA1c**, glycated hemoglobin A1c; **BMI**, body mass index; **SBP**, systolic blood pressure; **DBP**, diastolic blood pressure; **FBG**, fasting blood glucose; **TC**, total cholesterol; **TG**, triglyceride; **HDL-C**, high density lipoprotein cholesterol; **LDL-C**, low density lipoprotein cholesterol; **eGFR**, estimating glomerular filtration rate; **WBC**, white blood cell.

2. *, data were square transformed.

**Supplemental Table 2**. The correlation between fasting blood glucose, glycated hemoglobin A1c, body mass index, blood pressure, lipid profile, and estimated glomerular filtration rate in 6,349 Chinese adults

|  | Fasting blood glucose | Glycated hemoglobin A1c |
| --- | --- | --- |
| Glycated hemoglobin A1c | r=0.38 | N/A |
| Body mass index | r=0.19 | r=0.24 |
| Systolic blood pressure | r=0.29 | r=0.21 |
| Diastolic blood pressure | r=0.19 | r=0.16 |
| Total cholesterol | r=0.15 | r=0.19 |
| Total triglycerides | r=0.18 | r=0.16 |
| High-density lipoprotein cholesterol | r=-0.13 | r=-0.1 |
| Low-density lipoprotein cholesterol | r=0.16 | r=0.21 |
| Estimated glomerular filtration rate | r=0.001* | r=-0.17 |

**Note**: N/A, not applicable. All p<0.01 except that estimated glomerular filtration rate (p=0.9)

**Supplemental Table 3**. The mean and standard deviation at three time points across different trajectories of hs-CRP (mg/L)

| Time point | Low-stable | Moderate-increased | Moderate-fluctuated | High-decreased |
| --- | --- | --- | --- | --- |
| Sample size | 5,174 | 208 | 679 | 288 |
| 2013 | 0.56 (0.31, 1.02) | 1.78 (1.00, 2.64) | 0.65 (0.33, 1.32) | 5.36 (4.57, 6.68) |
| 2014 | 0.50 (0.30, 0.9) | 2.00 (0.90, 2.90) | 5.12 (5.12, 5.12) | 2.30 (1.30, 3.10) |
| 2015 | 0.70 (0.46, 1.10) | 5.62 (4.81, 7.45) | 0.68 (0.60, 1.33) | 2.90 (1.90, 3.52) |

**Supplemental Table 4**. Adjusted hazards ratios and 95% confidence intervals for risks of incident diabetes (2016-2018) across different trajectories of hs-CRP during 2013 and 2015 among 6,349 Chinese adults

| Model | Different change patterns of high sensitivity C-reactive protein | | | |
| --- | --- | --- | --- | --- |
|  | Low-stable | Moderate-increased | Moderate-fluctuated | High-decreased |
| n | 5,174 | 208 | 679 | 288 |
| Case # | 168 | 16 | 32 | 19 |
| Age- and sex-adjusted | **1 (ref)** | 2.18 (1.31, 3.64) | 1.4 (0.96, 2.05) | 1.94 (1.21, 3.12) |
| Multivariate-adjusted * | **1 (ref)** | 2.16 (1.23, 3.78) | 1.99 (1.28, 3.11) | 1.38 (0.83, 2.29) |

**Abbreviation**: **hs-CRP**, high sensitivity C-reactive protein.

* Adjusted for age, sex, the latest BMI (kg/m^2^), systolic blood pressure (mmHg), diastolic blood pressure (mmHg), total cholesterol (mmol/L), triglyceride (mmol/L), low-density-lipoprotein cholesterol (mmol/L), high-density-lipoprotein cholesterol (mmol/L), eGFR (ml/min/1.73m^2^), fasting blood glucose (mmol/L), and glycated hemoglobin A1c (%).
